# The North London Hospital for Consumption

**Published:** 1893-04-29

**Authors:** 


### FESTIVAL DINNERS.

### THE NORTH LONDON HOSPITAL FOR CONSUMPTION.

Mr. H. L. W. Lawson, M.P., presided at the annual festival of this charity at the Whitehall Rooms, Hotel Metropole. Amongst those present were Mr. A. Hoare (treasurer), Mr. J. D. Alcroft, Mr. C. Tyler, Colonel A. M. Arthur, the Rev. H. Hughes, Mr. J. S. Fletcher, the Rev. A. Godson, Dr. F. Weaver, Mr. W. F. Malcolm, Mr. T. F. Downes, Mr. H. C. Burdett, Dr. F. R. Walters, Dr. Douglas Pidcock, Dr. J. E. Squire, Dr. Watson, Mr. C. E. Graseman, Dr. Danford Thomas, Dr. Bernard Dyer, the Rev. T. Turner, Dr. Forbes Winslow, and Mr. L. H. Hill (secretary). The Chairman proposed the toast of the evening, "Prosperity to the North London Hospital for Consumption and Diseases of the Chest." He said they were there to aid in fighting the saddest and surest of all the diseases that afflict humanity, and he was glad to say that he had received many letters from various parts of the country expressing sympathy with their work. Among others he had received a letter from the late Lord Derby regretting his inability to attend, and referring to a contribution, which he said he hoped would compensate for his absence—one of the last acts of a truly English life being to help the hospital in whose aid they were met that night. London was a place peculiarly favourable to the development of this disease, and he thought he might say that the hospital was in the right place and was conducted on the right lines, and he hoped they would make a special effort this year to support the extension of the work. In response subscriptions were announced to the amount of £1,200.

